# Nanomaterials-mediated adenosine pathway inhibition for strengthening cancer immunotherapy

**DOI:** 10.7150/thno.108931

**Published:** 2025-03-31

**Authors:** Wenhu Zhu, Fei Wu, Zhanhong Qiao, Ming Zhao, Haiyang Hu

**Affiliations:** Department of Pharmaceutics, School of Pharmacy, Shenyang Pharmaceutical University, No. 103, Wenhua Road, Shenyang, 110016, P. R. China.

**Keywords:** adenosine, adenosine pathway inhibitors, nanomaterials, cancer immunotherapy, immunosuppression

## Abstract

Immunotherapy has developed into an attractive tumor treatment strategy. However, the existence of an immunosuppressive tumor microenvironment (ITME) greatly reduces the efficacy of immunotherapy. Adenosine (ADO) is one of the vital negative feedbacks in ITME, which inhibits antigen presentation and immune cell activation by binding to adenosine receptors (ADORs), thus tremendously suppressing immune response. Currently, the treatment effect of numerous inhibitors targeting the ADO pathway has been demonstrated in early clinical trials of various tumors. Nevertheless, the clinical application of these inhibitors is still plagued by diverse issues, such as short half-life, a single administration route, low bioavailability, etc. With the progress of nanotechnology, the delivery system of ADO inhibitors based on nanomaterials can solve the above problems. This review discusses the utilization of nanomaterials as a prospective method to inhibit ADO pathway and enhance immunotherapy outcomes. Specifically, the immunosuppressive mechanisms of ADO are summarized, and the corresponding intervention strategies are proposed. Then plentiful nanomaterials targeting the ADO pathway are highlighted, including phospholipids and polymers-based nanomaterials, mesoporous nanomaterials, biomimetic nanomaterials and metal-based nanomaterials. Finally, the outlook and challenges about nanomaterials-mediated ADO pathway inhibition were outlined, expecting to promote the clinical application of ADO inhibitor nanomedicines.

## Introduction

Immunotherapy has become a popular cancer treatment approach due to its strong specificity, wide applicability, and prevent tumor recurrence 1. However, the existence of ITME significantly restricts the effectiveness of tumor immunotherapy 2. ADO plays an immunosuppressive role in cancer therapy, which weakens the immune response 3-5. ADO is mainly produced by hydrolysis of ATP in the continuous action of Ectonucleoside triphosphate Diphosphohydrolase-1 (CD39) and Extracellular 5'-nucleotidase (CD73) 6-7. Under normal physiological conditions, the concentration of ADO ranges from 0.05 to 0.20 μmol/L 8, which has ability of anti-inflammatory effects, defends tissues from damage and stress as well as regulates ATP and ADP levels, and is essential for maintaining cellular energy balance and metabolism 9-10. Nonetheless, in the tumor microenvironment (TME), the ADO concentration increases to 1-10 mmol/L 11-12. On the one hand, dying tumor cells release a mass of ATP during Immunogenic cell death (ICD) after diverse treatments, which causes a raising in ADO, the hydrolysate of ATP. On the other hand, Hypoxia-inducible factor 1 (HIF-1) and Transforming growth factor β (TGF-β) accelerate high transcription and expression of CD39 and CD73 to promote ATP breakdown, which enhances ADO levels in the TME 13-17.

ADO interacts with ADORs to carry out immunosuppressive effects. Extracellular ADO activates cell signaling pathways through four known G-protein-coupled ADORs A1R, A2aR, A2bR, and A3R 18. Among them, A2aR and A2bR have shown an indispensable contribution to the immunosuppressive effect. A2aR is a high-affinity receptor expressed on multiple immune cells, while A2bR is principally expressed by macrophages and Dendritic cells (DCs) 19. ADO binding to A2aR/A2bR on DCs and macrophages poses a serious threat to their antigen presentation function. Moreover, different from the ADO released by normal cells under stress or injury, the extracellular ADO of tumor cells binds to A2aR on T lymphocytes to restrain the infiltration and function of T lymphocytes and induce the generation of ITME with plentiful immunosuppressive cells, such as Myeloid-derived suppressor cells (MDSCs), regulatory T cells (Tregs) and tumor-associated macrophages (TAMs) 20-23. Hence, restraining ADO signaling presents an unparalleled opportunity to remodel the ITME for effective tumor immunotherapy 24.

Based on the immunosuppression of the ADO pathway, restraining ADO production or blocking ADO binding to ADORs has become the primary strategy to relieve ADO immunosuppression and improve immunotherapy. At present, although multifarious inhibitors of blocking ADO production and ADORs antagonists have been applied in the preclinical or clinical development stage 25, these inhibitors have short half-lives and low bioavailability, which are more limited in clinical application.

In recent years, tumor immunotherapy has developed rapidly and extensively with the continuous progress of nanotechnology and material science 26-28. The corporation of nanotechnology and immunotherapy makes immunotherapy more effective and less toxic. So far, a wide variety of nanomaterials have been utilized in the development of tumor immunotherapy 29, such as polymers, mesoporous materials, and biomimetic nanomaterials. Nanomaterials exhibit increased drug-loading capacity and durable systemic circulation than conventional systems. Besides, nanomaterials enable the evasion of medicinal molecules from host immune system attacks and enzymatic breakdown. A variety of unique nanomaterials have been created to tackle the problems associated with ADO inhibitors, including short half-life, quick clearance, low bioavailability, and restricted administration routes, thereby offering expanded choices for drug delivery 30-31. Nanomaterials, including liposomes and inorganic nanoparticles, enhance the stability and bioavailability of small molecule ADO pathway inhibitors 32. For antibodies and siRNA, their complex structure and sensitivity to environmental factors necessitate the need for more specialized delivery vectors, such as biomimetic nanomaterials 33. Nanomedicines-mediated ADO inhibition can make up for the defects of traditional ADO pathway inhibitors and further raise the effect for tumor immunotherapy, so it is necessary to provide an overview of nanomedicine that inhibits ADO.

**Figure 1:**
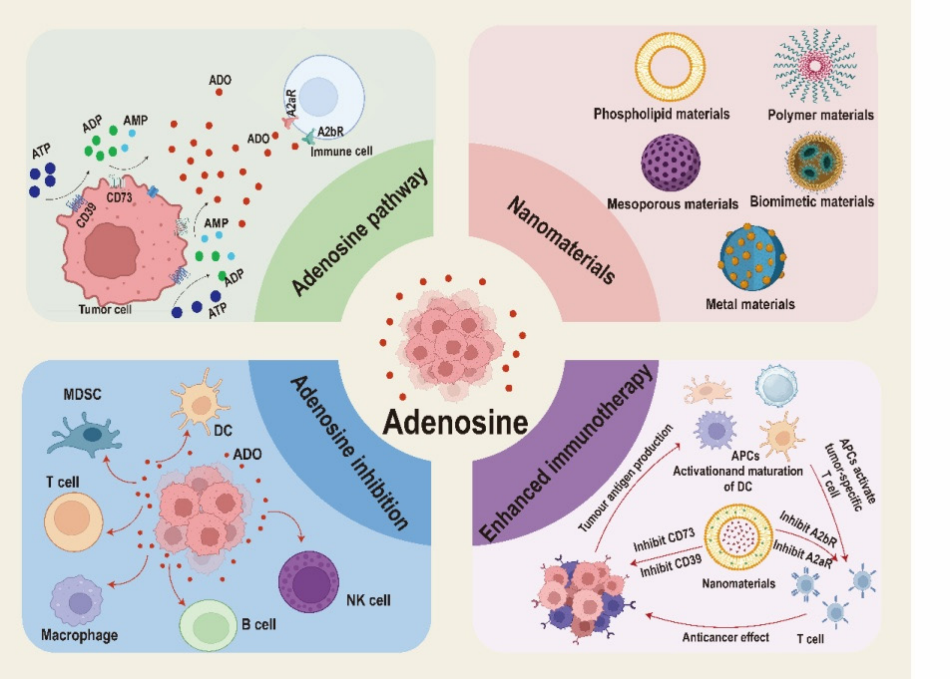
Schematic illustration of nanomaterials inhibited ADO pathway to enhance tumor immunotherapy. Created with BioRender.com.

In this review, we offer a comprehensive summary and in-depth discussion on the inhibition of ADO pathway by nanomedicines in tumor immunotherapy, focusing on the immunosuppressive mechanism of ADO and the application of nanomaterials targeting ADO pathway to boost immunotherapy (**Figure 1**). Firstly, we discuss the immunosuppressive mechanism of ADO in different cell types and intervention strategies. Then, we pay special attention to the strategies of combining various nanomaterials (including phospholipids and polymers-based nanomaterials, mesoporous nanomaterials, biomimetic nanomaterials and metal-based nanomaterials) with ADO pathway inhibitors to enhance tumor immunotherapy. Finally, we point out the development prospects and current challenges in the field of nanomedicines-mediated ADO pathway inhibition, aiming to further promote the clinical translation of ADO pathway inhibitors and enrich the clinical treatment options of tumor immunotherapy.

## Immunosuppressive mechanism ADO

**Figure 2:**
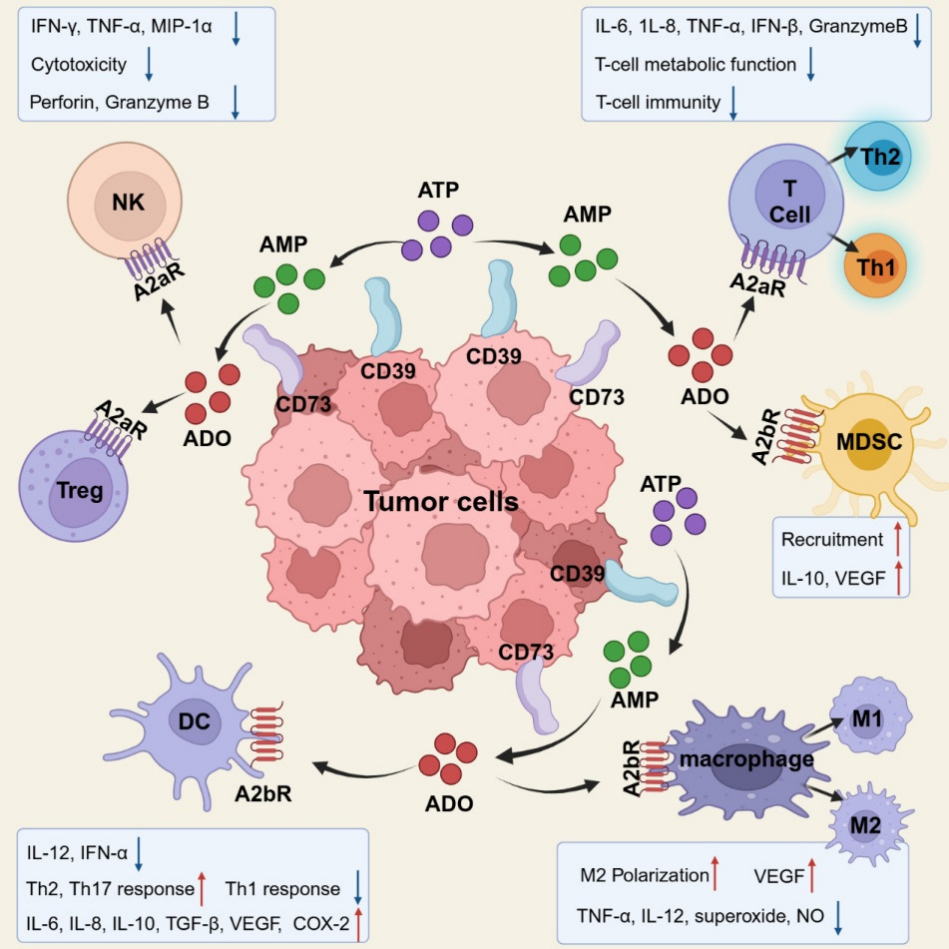
Immunosuppressive effects of ADO on various types of immune cells. Created with BioRender.com.

ADO causes immune suppression against tumors by binding to ADORs on various cells critical to the tumor immunological cycle and activating ADORs for distinct signaling pathways (**Figure 2**).

### Inhibit antigen presentation

As antigen-presenting cells (APCs), DCs play a critical role in immunological modulation and activation by initiating and regulating both innate and adaptive immune responses 34. DCs capture tumor-associated antigens and express co-stimulatory molecules, such as CD86 and CD80, forming peptide-MHC complexes that are presented to T lymphocytes to elicit an anti-tumor immune response 35. However, several research have demonstrated that ADO inhibits DC activation and antigen presentation. Within the TME, ADO influences the maturation and differentiation of DCs. Specifically, when ADO binds to its A2bR on DCs, it drives their differentiation into a distinct phenotype characterized by pro-angiogenic, immune-suppressive and tolerogenic phenotype 36. Unlike normal DCs, this aberrant DC phenotype suppresses the production of pro-inflammatory mediators, such as TNF-α and IL-12, while augmenting the secretion of pro-tumor proteins, including VEGF (Vascular endothelial growth factor), IL-6, TGF-β, IL-8, and IL-10 37. As a result, this impairs the activation of antigen-specific cytotoxic T lymphocyte (CTL) responses and hinders the differentiation of CD4^+^ T cells into anti-inflammatory Th1 cells, ultimately facilitating promoting tumor progression 38-39. Furthermore, ADO activates A2aR on DCs, leading to the downregulation of substances that stimulate MHC-II and CD86 expression, further compromising their antigen-presenting capacity. In conclusion, ADO interacts with DCs through A2aR and A2bR, inducing abnormal differentiation and suppressing tumor antigen presentation, which collectively inhibit T-cell activation 40.

### Inhibit T-lymphocyte activation

APCs form peptide-MHC complexes with antigens and present these complexes to lymphocytes, thereby activating the T-cell receptor (TCR) on their surface 41. Upon activation, the TCR associates with a cluster of differentiation 3 (CD3) to form a complex, which is subsequently phosphorylated by the lymphocyte-specific protein tyrosine kinase (LCK). This phosphorylation initiates signal transduction via immunoreceptor tyrosine stimulation patterns, facilitating the connection between zeta-chain-associated protein kinase 70 (ZAP-70) and CD3ζ, ultimately triggering T cells 42-43. Within the TME, however, ADO inhibits T lymphocyte activation. Specifically, ADO suppresses CD4^+^ T cell activation by downregulating MHC class II (MHC-II) expression on DCs. Additionally, elevated A2aR expression on T cells enables ADO to activate the cyclic AMP-protein kinase A-C-terminal Src kinase (cAMP-PKA-CSK) pathway 44. Upon pathway activation, CSK phosphorylates and deactivates LCK, reducing the tyrosine phosphorylation of TCR/CD3ζ chains following T cell activation, therefore attenuating TCR signal transmission 45. Furthermore, CSK activation suppresses interleukin-2 receptor (IL-2R) signaling in T lymphocytes, leading to the downregulation of the co-stimulatory molecule T-cell-specific surface glycoprotein (CD28), which further decreases the stimulation and growth of CD4^+^ T cells 46. Given that CD4^+^ T cells activation is essential for activating CD8^+^ T cells, this interconnected process also results in partial of suppression of CD8^+^ T cell activation. Moreover , ADO binding to A2aR activates the cAMP-PKA-cAMP response element-binding protein (CREB) pathway , wherein CREB inhibits nuclear factor kappa B (NF-κB) and nuclear factor of activated T cells (NFAT), resulting in a decrease in the secretion of anti-tumor cytokines such as IL-2, IL-4, IL-5, IL-6, IL-17, IFN-γ, and TNF-α, thereby inhibiting the activation of both CD4^+^ and CD8^+^ T cells 47.

### Inhibit T-lymphocyte transport and infiltration

The migration of effector T cells (Teffs) to the TME and their subsequent infiltration into tumor tissues are critical steps in initiating tumor immunotherapy. Upon reaching the tumor site via the bloodstream 48, Teffs, particularly CTLs, infiltrate the tumor and selectively recognize antigen peptide-MHC complexes on the tumor cell surface. This recognition forms an immunological synapse, triggering the release of perforin and granzymes, which mediate tumor cell elimination 49. However, the persistent accumulation of ADO within the TME inhibits the hampers chemotaxis and infiltration of specific T cells by binding to the A2aR on their surface 50. The transport of Teffs to the TME and their infiltration into tumor tissues are primarily mediated by the interaction between chemokines and their receptors 51, with C-X-C motif chemokine ligand 9 (CXCL9) and C-X-C motif chemokine ligand 10 (CXCL10) acting as key chemokines in this recruitment process. The expression of CXCL9 and CXCL10 is tightly linked to IFN-γ induction 52-53. Nevertheless, ADO binding to A2aR on Teffs activates the cAMP-PKA signaling pathway, suppressing IFN-γ secretion. This suppression reduces the intratumoral levels of chemokines such as CXCL9 and CXCL10, thereby impairing Teff recruitment and infiltration into the tumor site 54-55. In Caglar's breast cancer mouse model, blockade ADORs elevated IFN-γ-induced CXCL10 levels, promoted T lymphocyte recruitment, and suppressed tumor growth 56. Additionally, ion channels play a key role in regulating cytokine production and chemotactic responses in T lymphocytes 57-58. Specifically, in T lymphocytes, the KCa3.1 channel is critical for controlling chemotaxis and mediating ADO's inhibitory effects 59. ADO, via A2aR, stimulates cAMP production and activates PKA, which inhibits the KCa3.1 channel. This inhibition diminishes T lymphocyte chemotaxis, consequently suppressing their migration 60.

### Induce T-lymphocyte functional exhaustion

CD8^+^ T cells are key effector cells in anti-cancer immunotherapy 61. Upon infiltrating tumor tissues, T cells often enter a dysfunctional state known as T-cell exhaustion, which impairs their activity and proliferation, thereby facilitating tumor immune evasion. This condition primarily results from diminished effector cytokines within the TME, upregulation of inhibitory receptors, and immunosuppressive effects mediated by specific regulatory immune cells 62. Within the TME, ADO acts as a potent immunosuppressive factor. By binding to ADORs on immune cells, ADO induces T-cell functional exhaustion through distinct specific signaling pathways. Consequently, the regulation of T cell functional exhaustion by ADO can be classified into extrinsic and intrinsic mechanisms 63.

#### External pathway

In the extrinsic pathway of CD8^+^ T cell functional exhaustion within the TME 64, ADO primarily depletes T lymphocyte function by modulating tumor-associated immune cells. Various immune cells, including macrophages, natural killer cells (NKs), MDSCs, and Tregs, influence CD8^+^ T cell exhaustion by producing cytokines 65-66.

ADO production is a central mechanism by which Tregs suppress immune activity 67. Tregs convert extracellular ATP into ADO through the enzymatic activity of CD39 and CD73 expressed on their surface. Upon binding to the A2aR on Tregs, ADO enhances the function of Foxp3^+^ Tregs, promotes their proliferation, and upregulates the expression of PD-1 and CTLA-4 on their surface, thereby eliciting additional immunosuppressive responses 68-69. These effects include increased secretion of inhibitory cytokines such as IL-10 and TGF-β, and upregulation of CD39 and CD73 expression, which further amplifies ADO production. This reciprocal interplay between Tregs and the ADO pathway fosters a profoundly immunosuppressive environment within the TME, consequently limiting the efficacy of anti-tumor immune responses. Additionally, ADO exerts immunosuppressive effects via its interaction with A2bR on Tregs. Activation of A2bR stimulates the secretion of pro-angiogenic factors, such as VEGF, which supports tumor growth 70. In summary, ADO augments the immunosuppressive activity of Tregs, while Tregs, in turn, actively generate ADO, establishing a feedback loop that suppresses anti-tumor immune responses and supports tumor progression.

As innate lymphocytes, NKs regulate CD8^+^ T cell antiviral and antitumor immunity by secreting IFN-γ 71. However, ADO binds to A2aR to suppressing NKs maturation, inhibiting their proliferation, and reducing the proportion of mature NKs in the TME 72. Furthermore, ADO signaling, through crosstalk with the γ49D receptor, markedly restrains NKs cytotoxicity and the secretion of TNF-α, IFN-γ, GM-CSF, and MIP-1α, thereby contributing to CD8^+^ T cell exhaustion 73.

Research demonstrates that M2 macrophages suppress T lymphocyte function and promote T lymphocyte exhaustion, thereby facilitating tumor proliferation and metastasis 74. Within the TME, ADO drives macrophage polarization toward the M2 phenotype. ADO activates A2aR on macrophages, elevating intracellular cAMP levels. The increase in cAMP activates the cAMP-PKA and PI3K-PKC-HIF1 signaling pathways, which enhance the secretion of VEGF. Similarly, ADO activates A2bR on macrophages, promoting in increased VEGF secretion through the MAPK-AP-1 signaling pathway. Elevated VEGF levels further reinforce M2 macrophage polarization, contributing to T lymphocyte functional exhaustion 75-77. Additionally, upregulated cAMP levels activate the EPAC-p38-C/EBP signaling pathway, increasing IL-10 secretion and further driving macrophage polarization toward the M2 phenotype 78.

**Figure 3:**
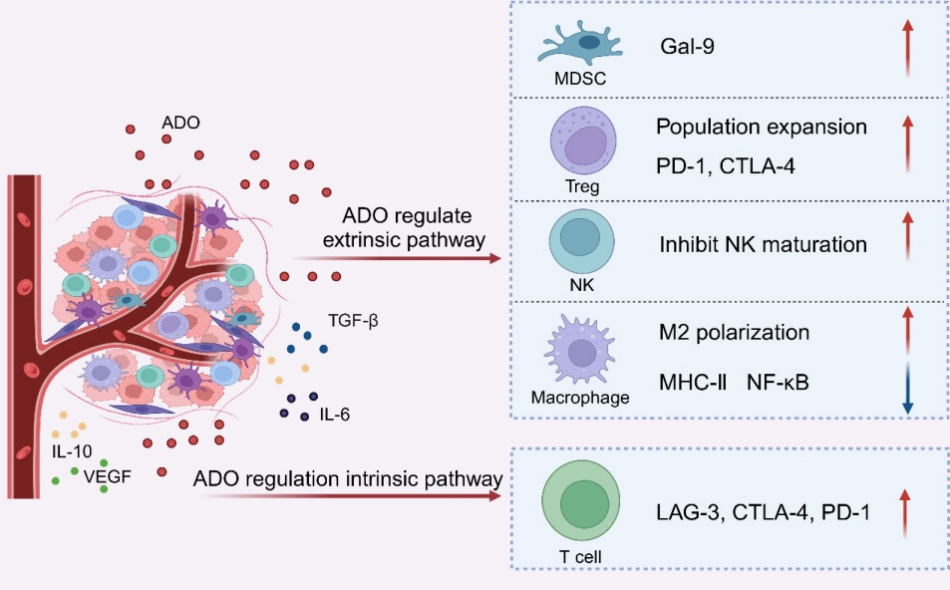
ADO propels T lymphocytes functional exhaustion through extrinsic and intrinsic regulatory mechanisms. Created with BioRender.com.

MDSCs induce functional exhaustion in CD8^+^ T cells and impair the tumoricidal activity and function of CTLs within the TME 79. Research has revealed that MDSCs from patients with myelodysplastic syndromes (MDS) exhibit high levels of galectin-9 (Gal-9). As a ligand for T cell immunoglobulin and mucin domain-containing protein 3 (TIM-3), Gal-9 exerts potent immunosuppressive effects. Upregulated in MDSCs via the AKT/mTOR signaling pathway, Gal-9 binds to the inhibitory receptor TIM-3 on CD8^+^ T cells, thereby promoting CD8^+^ T cell exhaustion 80-83. This immunosuppressive effect is further amplified by ADO signaling. In a melanoma mouse model, A2aR antagonists reduced MDSCs accumulation, restored CTLs functionality, and mitigated MDSC-mediated tumor growth and immunosuppression 84. Conversely, in a lung carcinoma mice model, A2bR agonists promoted the differentiation of MDSCs into the granulocyte subset, exacerbating CD8^+^ T cell functional exhaustion 85 (**Figure 3**).

#### Internal pathway

The hallmark of T lymphocyte functional exhaustion is the overexpression of inhibitory receptors. These receptors impair T cell function through multiple mechanisms, including the upregulation of genes that suppress T cell activity, competing with co-stimulatory receptors for ligand binding to prevent the formation of micro-clusters and lipid rafts, and modulation T cell motility 86-88. ADO decreases the capability of CTLs to eradicate tumors. It depletes T lymphocytes via intrinsic pathways, promoting tumor immune evasion by upregulating inhibitory receptor expression. Specifically, ADO enhances the expression of immune checkpoint proteins, such as LAG-3, CTLA-4, and PD-1 by activating A2aR on T lymphocytes. This activation leads to increased inhibitory receptor expression, suppression of T lymphocyte function, and diminished immunogenic stimulation 89. In the MC38-OVA tumor-bearing mouse model, administration of ADORs agonists markedly exacerbated PD-1 levels and mediated T lymphocyte functional exhaustion. ADO inhibitors reduced PD-1 expression and alleviated T lymphocyte functional exhaustion 90. The binding of ADO to its receptors enhances the gene expression of immunological point receptors through intrinsic pathways, driving T cell functional exhaustion (**Figure 3**).

## Advantages of targeting the adenosine pathway

Immunotherapy is widely regarded as one of the most promising modalities for cancer treatment, with substantial clinical advances in recent years. Among these approaches, immune checkpoint blockade (ICB) targeting PD-1, PD-L1, and TGF-β has exhibited remarkable therapeutic efficacy across various solid tumor models, bolstering confidence among researchers 91,92. Notably, PD-1/PD-L1 immune checkpoint inhibitors has been the most extensively investigated in recent years 93. As of October 2024, there are 125 PD-1/PD-L1 inhibitors under development globally, with 93 (74%) in early-stages development, encompassing preclinical to Phase I trials. Additionally, 13, 14, 1, 2, and 2 products are in Phase I/II, Phase II, Phase II/III, Phase III, and approved stages, respectively. Despite the considerable therapeutic benefits of targeting the PD-1/PD-L1 axis, including robust efficacy in numerous cancers and relatively low toxicity compared to other immunotherapies 94,95-only 20-30% of patients respond to PD-1/PD-L1 monotherapy. Moreover, its efficacy is limited in tumors with low T-cell infiltration, such as pancreatic cancer. To broaden the reach of immunotherapy and benefit a larger population, there is an urgent need to identify and target novel immune checkpoints, paving the way for next-generation cancer immunotherapies.

Compared to well-established immune checkpoints such as PD-1/PD-L1 and CTLA-4, the ADO pathway has emerged as a prominent focus in cancer immunotherapy due to its distinct advantages. These advantages arise primarily from the multifaceted immunoregulatory roles of the ADO pathway and the unique features of the TME, positioning it as a compelling target for novel therapeutic strategies 96.

The ADO pathway exerts broad, multi-target immunoregulatory effects. Under hypoxic conditions, tumor cells and tumor-associated immune cells, such as Tregs and MDSCs) release ATP, which is subsequently converted extracellularly into ADO by CD39 and CD73 97. ADO binds to A2aR and A2bR on effector immune cells, inhibiting T cell proliferation, NKs cytotoxicity, and DCs antigen presentation while simultaneously enhancing the suppressive function of Tregs and amplifying the immunosuppressive activity of MDSCs 98. This pathway predominantly modulates immunosuppressive cells within the TME and targets elevated local ADO concentrations in tumors rather than the systemic immune system, conferring greater tumor specificity. Furthermore, ADO broadly suppresses the functions of multiple immune cell types 99.

In contrast, PD-1/PD-L1 inhibition primarily targets PD-1 on T cells and its ligand, PD-L1, to reinvigorate exhausted T cell activation, thereby enhancing tumor eradication 100. CTLA-4 inhibition works by modulating the activation of naïve T cells through competitive binding with B7 molecules, thereby suppressing immune responses 101. TGF-β exerts its effects by suppressing effector immune cells, inducing immunosuppressive cell populations, and promoting tumor proliferation and metastasis 102. Compared to the ADO pathway, these conventional immune checkpoints exhibit more narrowly defined regulatory mechanisms.

Notably, the ADO pathway demonstrates superior efficacy in addressing "immune-cold" tumors, which are characterized by low T cell infiltration 103. Inhibition of the ADO pathway enhance T cell infiltration, thereby overcoming the limitations of PD-1/PD-L1 inhibitors in these settings. Furthermore, ADO pathway inhibitors exhibit lower toxicity and reduced resistance compared to PD-1/PD-L1 and CTLA-4 inhibitors 104. As ADO pathway targets are predominantly overexpressed in the TME, their inhibition exerts minimal effects on the systemic immune system. Additionally, the mechanism of action of ADO pathway inhibitors is distinct from PD-1/PD-L1 and CTLA-4 pathways, circumventing cross-resistance issues commonly associated with other checkpoint inhibitors.

Consequently, the ADO pathway holds represents a complementary strategy to conventional immune checkpoint inhibitors, particularly for tumors exhibiting robust immunosuppressive features or resistance to existing therapies, thus providing a novel avenue for advancing cancer treatment.

## Intervention strategies for targeting ADO

The ADO signaling pathway mediates antineoplastic immune suppression through CD39, CD73, and A2aR. Upregulation for conciseness of CD39 and CD73 promotes the proliferation of MDSCs and Tregs within tumor tissues, enhances the hydrolysis of ATP produced by tumor cells, increases the synthesis of immunosuppressive ADO, and facilitates immune evasion by tumor cells. The binding of ADO to A2aR represents a pivotal mechanism by which the ADO signaling pathway induces immune suppression. Consequently, targeting ADO production and blocking ADORs binding have emerged as key strategies for inhibiting this pathway 105. Numerous ADO-targeted inhibitors, including monoclonal antibodies and small-molecule inhibitors, are currently under evaluation in clinical trials to advance antineoplastic immunotherapies (**Table 1**). Preliminary findings demonstrate that ADO inhibitors targeting molecules such as CD39, CD73, and A2aR to reduce ADO production and prevent its binding to ADORs. Although still in the early stages of clinical development and not yet widely implemented, directly suppress tumor cell proliferation and dissemination, thereby improving patient survival and quality of life 106.

### Targeted CD39-CD73 ADO inhibitors

In numerous tumor immunotherapies, anti-CD39 antibodies have been shown to regulate CD39 enzyme activity on tumor cell surfaces, effectively inhibit internal metastasis 107. In a lung cancer model, an anti-CD39 antibody was demonstrated to upregulate CD107a expression on infiltrating NKs, promoting the release of IFN-γ and thereby enhancing the cytotoxicity against cancer cells 108. ES014, a bispecific antibody targets both CD39 and TGF-β, has been found to simultaneously suppress CD39 enzyme activity and neutralize autocrine and paracrine TGF-β, targeting two major immunosuppressive pathways within the TME. As a result, ES014 enhances antineoplastic immunogenicity by increasing extracellular inflammatory ATP levels and reducing the accumulation of ADO and TGF-β within the TME 109. In studies of anti-CD73 antibodies, Kellner et al. reported that the anti-CD73 antibody 22E6 selectively inhibits the activity of vesicles formed by the CD73 interactions with tumor cell membranes 110. In experimental mouse models of acute lymphoblastic leukemia (ALL), administration of 22E6 delayed initial tumor growth in some subjects. Consequently, ALL cells from treated mice exhibited reduced CD73 expression, suggesting impaired tumor immune evasion. Additionally, Pe et al. demonstrated that IPH5201 (anti-CD39 monoclonal antibody) and IPH5301 (anti-CD73 monoclonal antibody) selectively target both the membrane-bound and soluble forms of CD39 and CD73, thereby preventing the conversion of immunogenic ATP into immunosuppressive ADO 111. IPH5201 and IPH5301 bolster antineoplastic immunity by activating DCs and macrophages, thus restoring the activation of T cells in cancer patients.

Research on inhibitors targeting CD39 and CD73 has extensively investigated several small-molecule inhibitors. For example, ESoo2033, a CD39 inhibitor, stabilizes extracellular anti-inflammatory ATP and disrupts the synthesis of immunosuppressive ADO within the TME, thereby restoring anti-tumor immunity 112. Additionally, Dana et al. have developed a novel CD73 inhibitor, AB680, which enhances T cell proliferation, cytokine release, and tumor cytotoxicity. AB680 has advanced to phase I clinical trials for further evaluation 113.

### Targeted A2aR ADO inhibitors

Inhibition of A2aR activation enhances antineoplastic immunogenicity 114. A2aR inhibitors bolster the effector functions of CTLs and prevent the polarization and recruitment of immunosuppressive lymphocytes within the TME, thereby augmenting antineoplastic efficacy 115. AZD4635, a novel oral ADO inhibitor, enhances antitumor immunity by promoting antigen presentation and restoring the function of Teffs 116. In a Phase I trial for resistant tumors, the combination of AZD4635 with anti-PD-L1 monoclonal antibodies significantly improved anticancer immunotherapy outcomes 117. However, in some patients, insufficient immune cells infiltration or mutations and deletions in cancer cell antigens may impair TCR recognition of targets, thereby reducing the efficacy of certain A2aR inhibitors. Nevertheless, these inhibitors can effectively enhance antitumor effects when combined with chimeric antigen receptor T-cell (CAR T) therapy or other drugs 118. CPI-444 exhibits markedly increased efficacy when paired with anti-PD-1/PD-L1 and anti-CTLA-4 antibodies 119. In 2020, Fong et al. reported a clinical trial involving 68 patients with advanced renal cell carcinoma (RCC), where 17% of the 33 RCC patients treated with CPI-444 monotherapy achieved disease control for over six months, compared to 35% of the 35 patients receiving CPI-444 combined with atezolizumab 120.

## Nanomaterials targeting the ADO pathway in tumor immunotherapy

ADO inhibitors, by blocking key receptors in the ADO signaling pathway, such as A2a and A2b, substantially mitigate the immunosuppressive state within the TME, positioning them as a potential breakthrough in cancer immunotherapy. However, their clinical application is constrained by challenges including short half-life, rapid clearance, low bioavailability, and restricted administration routes. These limitations arise primarily from their chemical properties, metabolic pathways, and pharmacokinetic distribution characteristics.

Currently, most ADO inhibitors in clinical development are small-molecule compounds. Their high-water solubility or lipophilicity vulnerable them susceptible to rapid metabolism by hepatic enzymes, particularly the cytochrome P450 system, significantly shortening in vivo duration of activity. Moreover, these drugs often exhibit low plasma protein binding affinity, increasing their susceptibility to renal clearance, which further accelerates the reduction in plasma drug concentration and contributes to their low bioavailability 121,122. For example, Park et al. highlighted these challenges in a pharmacokinetic study of SCH58261 in rats 123. Following intravenous administration (1 mg/kg) and oral administration (5 mg/kg), plasma concentrations of SCH58261 were measured, and pharmacokinetic parameters were determined using non-compartmental analysis (WinNonlin). The study revealed that SCH 58261 was nearly undetectable in plasma after oral administration, and its plasma concentration declined markedly within ten minutes of intravenous injection, resulting in a bioavailability of only 0.03%.

Another obstacle to the clinical translation of ADO inhibitors is their systemic distribution. ADO fulfills critical physiological roles in normal tissues, including inflammation regulation and cardiovascular protection. Systemic distribution of ADO inhibitors may result in off-target effects and systemic toxicity. Furthermore, ADO inhibitors are designed to target regions of high ADO expression within the TME, although systemic administration often fails to achieve precise localization to these areas 124.

These factors result in to suboptimal therapeutic efficacy and adverse effects, constraining the clinical potential of ADO inhibitors in cancer immunotherapy. Consequently, addressing these challenges are essential for the successful clinical translation of ADO inhibitors.

For instance, nanocarriers such as liposomes and polymeric nanoparticles encapsulate ADO inhibitors, effectively protecting them from metabolic enzyme systems and significantly extending their circulatory half-life in circulation 125. Moreover, nanoparticles with sizes typically ranging from 10 and 200 nm selectively accumulate at tumor sites through the enhanced permeability and retention (EPR) effect, thereby evading rapid renal clearance 126. Additionally, nanomaterials enhance the solubility of ADO inhibitors by modifying their surface chemistry-such as attaching hydrophilic molecules or polymers, thus improving absorption efficiency. Nanocarriers also shield ADO inhibitors from gastrointestinal degradation and first-pass hepatic metabolism, resulting in enhanced systemic bioavailability. To address the challenge of precise targeting within the TME, nanomaterials can be functionalized to facilitate targeted delivery of ADO inhibitors to the TME, reducing off-target distribution in normal tissues and reducing systemic toxicity. Furthermore, stimuli-responsive nanomaterials, such as those sensitive to pH, temperature, or enzymes, enable controlled drug release tailored to TME characteristics, maintaining elevated concentrations at the target site while limiting release in non-target areas.

The integration of ADO inhibitors with nanomaterials not only addresses the pharmacokinetic limitations of conventional small-molecule drugs but also augments the efficacy of tumor-specific immunotherapy. Furthermore, nanomaterials facilitate combinatorial strategies by co-delivering ADO inhibitors with other immune checkpoint inhibitors, thereby enhancing the therapeutic impact of ADO pathway inhibition in cancer immunotherapy. The subsequent sections will provide a comprehensive overview of various nanomaterials targeting the ADO pathway and their applications in tumor immunotherapy.

### Phospholipid and polymer-based nanomaterials

Small-molecule drugs targeting the ADO pathway have garnered considerable attention due to their distinct advantages, including rapid absorption and distribution, high bioavailability, robust stability, and sustained therapeutic effects 127,128. However, the complex physiological environment poses challenges for these drugs, such as limited solubility and difficulties in penetrating biological membranes. Liposomes are widely recognized for their excellent targeted delivery capabilities, excellent biocompatibility, and reliable safety profiles 129-134. Leveraging engineered surface functional groups, favorable biocompatibility, and controlled release properties, liposomes can encapsulate or adsorb small molecules onto their surface or within their structure, ensuring robust drug protection. Moreover, surface modifications or ligand conjugation of liposomes can enhance efficient drug delivery and facilitate targeted release, positioning them as a promising strategy for improving the therapeutic efficacy of small molecules in tumor immunotherapy.

Fu et al. developed a drug formulation designed to suppress the in-situ growth of melanoma models, as well as lung metastasis and subsequent recurrence 135. Given that POM-1, a CD39 inhibitor, may induce non-specific effects on the central nervous system upon systemic administration, the researchers engineered a lipid nanoparticle platform (POL) to overcome this limitation. This platform encapsulates an ICD inducer--oxaliplatin prodrug (OXA-C)--alongside POM-1. The POL system demonstrates high specificity, efficiently delivering POM-1 to tumor sites while minimizing off-target effects on normal cells. Upon reaching the tumor site during melanoma immunotherapy, POM-1 dissociates from POL due to weak electrostatic interactions and directly modulates the ATP-ADO pathway. The remaining lipid nanoparticles containing OXA-C penetrate deeply into tumor tissue and are taken up by tumor cells. Under the influence of intracellular lysosomes, these nanoparticles release OXA, thereby inducing the ICD effect in tumor cells. Furthermore, both in vitro and in vivo studies of POL revealed its robust efficacy in tumor suppression without notable toxic side effects. POL significantly enhances DCs maturation, promotes CD8^+^ T cell activation, reduces the number of myeloid cells, and markedly decreases tumor immunosuppressive factors. This dual approach not only amplifies the immune response while mitigating immunosuppression, effectively curbing the in situ proliferation of melanoma and reducing lung metastasis, but also addresses the challenges associated with ADO inhibitors in clinical applications, thereby enhancing the therapeutic potential of targeting the ADO pathway in tumor immunotherapy. Lipid nanomaterials exhibit biodegradability and biocompatibility, enabling efficient drug encapsulation and protection.

Liu et al. designed a lipid-based nanomaterial, RC-nIMs, incorporating R837 and caffeine (an A2aR antagonist) 136. This nanocarrier significantly enhances the therapeutic efficacy of combined radiotherapy (RT) and ensures efficient delivery of caffeine to tumor sites, overcoming the challenge of incomplete caffeine delivery.

**Figure 4:**
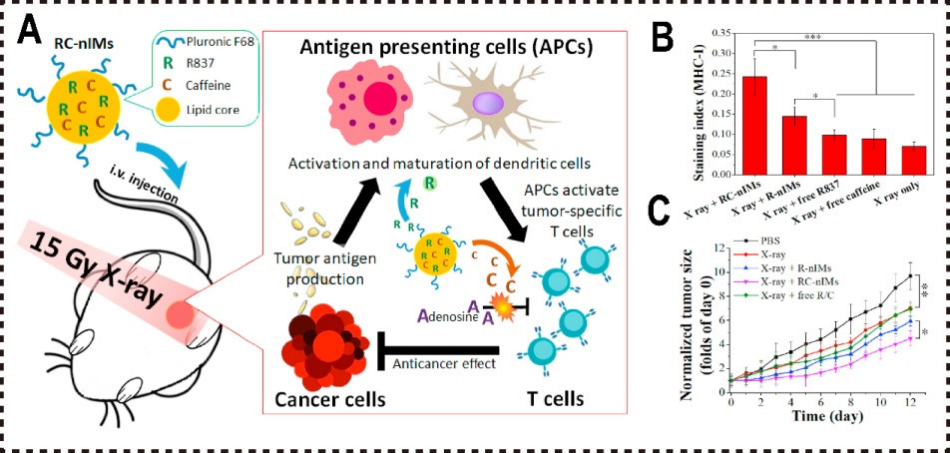
Phospholipid nanomaterials inhibited ADO pathways. (A) Schematic illustration of RC-nIM competitively inhibiting ADOR to block ADO pathway and enhance tumor immunotherapy synergistically with RT. (B) MHC-I staining ratio determined by flow cytometry (n = 5). (C) Growth curves of mice tumors in different treatment groups. Reproduced with permission from Ref. 136. Copyright 2020, Elsevier.

RT induces ICD in tumor cells, increasing tumor antigen release and, in synergy with R837, activates APCs, thereby amplifying immune responses. Furthermore, as an ADO analog, caffeine competitively binds to to A2aR on immune cells, mitigating ADO-mediated immunosuppression and effectively inhibiting the ADO pathway. In combination with R837, caffeine promotes macrophage polarization toward the M1 phenotype. Both in vitro and in vivo studies demonstrated that RC-nIMs loaded with R837 and caffeine efficiently induce M1 macrophage polarization and activation, enhance the infiltration of T cells and APCs within the tumor microenvironment, and suppress tumor growth (**Figure 4A**).

Polymer micelles are extensively investigated as drug delivery carriers due to their distinct physicochemical properties, which overcome the solubility limitations of poorly soluble drugs and extend the circulatory half-life of chemotherapeutic agents 137,140. They exploit the EPR effect to promote passive drug accumulation at tumor sites, thereby elevating drug concentrations within tumor tissues 141,142. Owing to their small particle size, polymer micelles provide notable advantages over other drug carriers in tumor targeting, enhancing both tumor tissue penetration and drug release efficiency 143.

In contrast to liposomes, which rely on hydration and lipid-water interactions for drug loading--often resulting in constrained drug loading capacity--polymer micelles can typically accommodate higher drug concentrations, markedly enhancing drug delivery efficiency. Furthermore, by adjusting the polymer structure, such as molecular weight and hydrophilic/hydrophobic ratio, drug loading and release profiles can be tailored to meet diverse therapeutic requirements. Polymer micelles can also be functionalized by conjugating polymer chains, such as grafted polyethylene glycol (PEG), to minimize macrophage recognition, thereby extending their circulatory half-life. This attribute is particularly critical for drugs necessitating prolonged treatment, such as ADO inhibitors, as it ensures more stable and sustained therapeutic effects.

**Figure 5:**
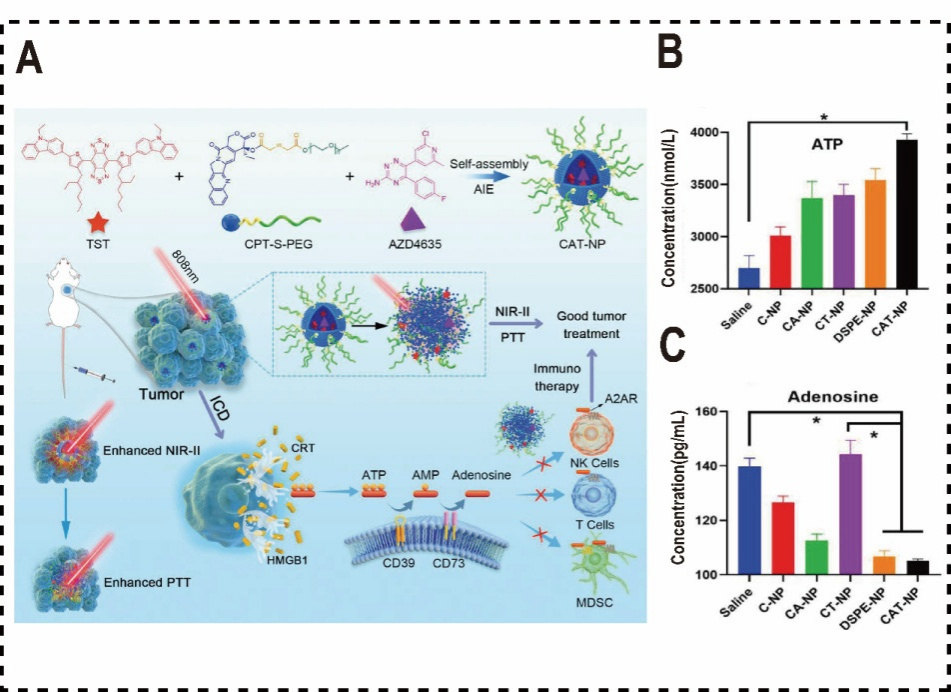
Polymer nanomaterials inhibited ADO pathways. (A) Diagram of CAT-NP's role in augmenting tumor immunotherapy through the inhibition of ADO binding to ADOR on immune cells. (B) The concentration of ATP in the TME. (C) The concentration of ADO in the TME. Reproduced with permission from Ref. 144. Copyright 2022, Wiley-VCH.

Wang et al. synthesized a novel aggregation-induced emission (AIE) molecule, TST, which endowed with AIE properties and photothermal therapeutic capabilities. They engineered a polymer micelle for drug delivery, co-assembled from the camptothecin prodrug (CPT-S-PEG) and the immune checkpoint inhibitor AZD4635, and TST using a nanoprecipitation method 144. CPT is conjugated to PEG via an esterification reaction, forming an amphiphilic compound (CPT-S-PEG). The resulting polymeric micelle, CAT-NP, significantly enhances the solubility of AZD4635 and overcomes its limited efficacy as a monotherapy by amplifying therapeutic outcomes when combined with other agents. This strategy not only significantly improves the solubility of AZD4635 but also addresses its limited efficacy as a monotherapy by enhancing therapeutic outcomes when combined with other drugs. Upon internalization by tumor cells, the redox-sensitive CPT-S-PEG degrades, triggering CAT-NP disassembly. The released TST promotes non-radiative decay and accelerates photothermal conversion, elevating tumor site temperature at the tumor site, which induces ICD in tumor cells and releases a substantial of ATP into the TME to recruit immune cells. The amphiphilic properties of polymer micelles enhances the solubility of the poorly soluble drug AZD4635, facilitating its efficient incorporation into CAT-NPs. Following CAT-NPs disassembly, the released AZD4635 competitively inhibits the binding of ADO to A2AR in a competitive manner, reversing ADO-mediated immunosuppression. This enhances effector T-cell proliferation o and increases the secretion of cytokines such as IFN-γ and TNF-α, thereby bolstering T cell responses to tumor antigens. Consequently, this strategy synergistically enhances the efficacy of chemotherapy and immunotherapy in tumors. In vivo anti-tumor experiments in mice, CAT-NP also exhibits robust anti-tumor efficacy, with CAT-NP inducing the highest levels of apoptosis and necrosis in cancer cells compared to other treatment groups (**Figure 5A**). To further mitigate the immunosuppressive effects of ADO on tumor immunotherapy, Yan et al. developed a redox-responsive polymer micelle nanovaccine for use in combination with the A2aR antagonist SCH58261 145. These micelles were formulated as an effective in situ vaccine by loading the ICD inducer doxorubicin (DOX) and the adjuvant TLR7/8 agonist R848. Within the TME, the elevated glutathione (GSH) concentrations in tumor cells trigger micelle disassembly, releasing DOX and R848 to induce ICD, stimulate DCs, and initiate an immune response. Concurrently, the A2aR antagonist SCH58261 functions as an ICB, inhibiting the ADO pathway in the TME, activating NKs and CD8^+^ T cells, and suppressing regulatory Tregs, thereby alleviating the ADO-mediated immunosuppression on tumor immunotherapy.

### Mesoporous nanomaterials

Mesoporous materials encompassing metal-based, carbon-based, silicon-based, and organic variants, are widely utilized in industrial catalysis, biomedicine, and smart sensors applications. However, their application as nanocarriers in drug delivery systems is constrained by significant limitations, such as poor biocompatibility, elevated toxicity, and difficulties in regulating degradation kinetics. Only select mesoporous inorganic materials, such as silica 130-131, 146, 147, certain metal oxides 132-133, 148, 149, carbon 134-135, 150, 151, metal-organic frameworks (MOFs) 136-138, 152-154, and hydroxyapatite 139-141, 155-157, have demonstrated potential for drug delivery applications. Compared to liposomes and polymeric materials, mesoporous materials typically exhibit superior drug loading capacities, making them particularly suitable for encapsulating drugs with higher molecular weights or greater hydrophobicity, such as ADO inhibitors. This enhanced drug loading capacity improves drug delivery efficiency. In contrast, liposomes are susceptible to drug leakage due to the phospholipid bilayer rupture, while polymer micelles may experience instability arising from compatibility challenges. Mesoporous materials, however, can achieve greater stability through modifications to pore properties and surface chemistry, thereby ensuring efficient drug delivery and controlled release. Additionally, mesoporous materials like silica exhibit excellent biocompatibility and low toxicity, providing a distinct advantage in drug delivery systems.

The primary pathway for ADO production involves the degradation of substantial ATP released by tumor cells via CD39 and CD73, resulting in ADO accumulation that suppresses immune cell functions. Hypoxia serves as another pivotal factor driving elevated ADO concentrations within the TME 158. Under hypoxic conditions, HIF-1 is activated in tumor cells. As a key regulator of genes encoding ADO metabolism-related enzymes, HIF-1 upregulates the activity of CD39 and CD73, thereby enhancing ADO production. Furthermore, HIF-1 induces the expression of A2bR, amplifying ADO signaling 159.

Building on this finding, Zhou et al. developed a macrophage-membrane-coated mesoporous silica nanoparticle platform loaded with DOX, R848, and catalase (CAT) to mitigate hypoxia and alleviate immunosuppression within the TME, thereby enhancing anti-tumor immunotherapy 160. As a supplemental oxygen (O_2_) source, CAT specifically catalyzes the decomposition of elevated endogenous hydrogen peroxide (H_2_O_2_) in tumor cells into O_2_, which inhibits the A2aR pathway in situ and downregulates the immunosuppressive function of Tregs. However, CAT is inherently unstable. In this design, mesoporous silica serves as a carrier with a large surface area and tunable pore sizes, enabling effective adsorption and immobilized on its surface and within its pores. This significantly increases CAT loading capacity and enhances its stability, as the porous structure and high chemical stability of silica confer protective effects. Additionally, the excellent biocompatibility of mesoporous silica preserves CAT activity. Characterization studies confirmed that silica augments CAT enzymatic activity. In vitro and in vivo experiments demonstrated that hypoxia reversal effectively suppresses the ADO pathway, offering a robust strategy to enhance tumor immunotherapy. The macrophage membrane coating enables active targeting of tumor sites, where the released CAT inhibits A2aR signaling pathway in Tregs, boosting the reactivity of tumor-specific CD8^+^ T cells. Concurrently, the released DOX and R848 induce ICD in tumor cells, activating DCs and further amplifying anti-tumor immunity.

MOFs have attracted significant interest in drug delivery applications. When engineered at the nanoscale, nanoscale MOFs (NMOFs) serve as highly effective nanocarriers for chemotherapy, photothermal or photodynamic therapy, and imaging 161-165. Compared to other materials, MOFs offer several distinct advantages: (1) their high porosity and expansive surface area enable a superior drug-loading capacity; (2) their chemical and physical properties can be readily tailored by incorporating organic groups or natural ligands. For example, MOFs containing lanthanide elements can emit fluorescence under ultraviolet (UV) excitation, with functional groups integrated for assertiveness into organic ligands through either pre-synthetic design or post-synthetic modification 166,167; (3) substrate materials can interact with encapsulated drug molecules via pores and open channels; (4) coordination bonds render MOFs biodegradable, (5) the structural features of MOFs facilitate the study of host-guest interactions, collectively position MOFs as promising candidates for in vivo administration drug administration and cancer therapy 168.

Adenosine kinase (ADK) is the primary enzyme responsible for the phosphorylating ADO into AMP, playing a pivotal role in regulating of ADO metabolism. Under normal physiological conditions, ADK's catalytic activity remains unaffected by low ADO concentrations 169. However, excessive accumulation within the TME can bind to ADK's active site, inhibiting its activity. Inorganic phosphates can restore ADK's catalytic function by forming ternary complexes with ADK and ADO, effectively displacing ADO from the active site 170,171. Leveraging this mechanism, Tang et al. developed calcium phosphate-reinforced MOFs (CaP@Fe-MOFs) to reduce ADO accumulation in the TME and mitigate ADO-induced immunosuppressive responses 172. In an acidic environment, inorganic phosphates regulate ADK activity in the presence of elevated ADO levels. To assess whether phosphate released from CaP@Fe-MOF influences ADK-mediated ADO phosphorylation, levels of ATP, ADP, AMP, and ADO were quantified under acidic and neutral conditions using high-performance liquid chromatography (HPLC). The results demonstrated that ADK catalyzes ADO phosphorylation to produce ADP and AMP. HPLC spectra revealed that under acidic conditions, CaP@Fe-MOF supplementation significantly enhanced the peak intensities of ADP and AMP. This suggests that CaP@Fe-MOF activates ADK, effectively suppressing the ADO pathway. In mouse tumor treatment studies, CaP@Fe-MOFs restored ADK activity via phosphates, promote ADO phosphorylation, and reduced ADO levels. Additionally, the iron component facilitated Fenton reactions to generate oxygen, further inhibiting ADO production. Both in vivo and in vitro experiments with CaP@Fe-MOFs confirmed their efficacy in alleviating ADO-mediated immunosuppression, offering novel insights into strategies for blocking ADO-mediated immune suppression and presenting a promising approach for tumor immunotherapy.

### Biomimetic nanomaterials

Biomimetic nanocarriers are an emerging class of nanomaterials designed to emulate the structure, function, and properties of natural biological systems. These carriers demonstrate excellent cellular compatibility, precise targeting specificity, extended circulatory longevity, and reduced immunogenicity 157-162, 173-178. However, most ADO inhibitors suffer from no-specificity, which impairs precise targeting and delivery to tumor sites. To address this limitation, the deployment of biomimetic nanomaterials for delivering ADO inhibitors has emerged as a promising strategy, with the potential to enhance the efficacy of tumor immunotherapy 179, 180. Compared to mesoporous, lipid-based, and polymeric nanomaterials, biomimetic nanomaterials offer distinct advantages in ADO inhibitor delivery, including superior biocompatibility, biodegradability, enhanced targeting precision, excellent transmembrane penetration, controlled drug release, high drug-loading capacity, and versatile surface functionalization. These attributes position biomimetic nanomaterials a highly promising platform for drug delivery, significantly improving the efficiency and therapeutic outcomes of ADO inhibitors.

Siriwon et al. employed CAR-T cells as carriers to deliver cross-linked multilayer liposome vesicles (cMLVs) loaded with SCH-58261, facilitating deep infiltration of T cells into the TME 181. Both in vivo and in vitro experiments demonstrated that CAR-T cells acting as active chaperones, enabled cMLVs to penetrate the ITME, effectively suppressing the ADO pathway and enhancing CAR-T cells therapy efficacy. Research has shown that ADO concentrations in tumor tissues markedly increase following photothermal therapy (PTT), partially diminishing the effectiveness of tumor immunotherapy. To address this, Zhao et al. developed a novel biomimetic photothermal nanomedicine that specifically inhibit CD73, thereby blocking ADO production and more effectively modulating the ITME 182. This nanomedicine was formulated using the photothermal agent black phosphorus quantum dots (BPQD) and the selective CD73 inhibitor α, β-methylene adenosine diphosphate (AMPCP), encapsulated within chitosan nanogels and coated with erythrocyte membranes (EM) featuring an AS1411 aptamer-simulated coating (AptEM@CBA). The AS1411- mediated targeting mechanism and EM-enhanced circulatory longevity time promoted the accumulation of the nanomedicine at tumor sites. PTT induced DCs maturation. While the concurrent release of AMPCP inhibited ADO production via CD73 blockade, mitigating ADO-mediated damage to DCs and inhibiting Tregs, thereby activating T lymphocytes. Beyond suppressing the growth of primary implanted tumors, the combination of CD73 blockade and PTT significantly amplified the antineoplastic immune response, contributing to the prevention the development of distant tumor development.

**Figure 6:**
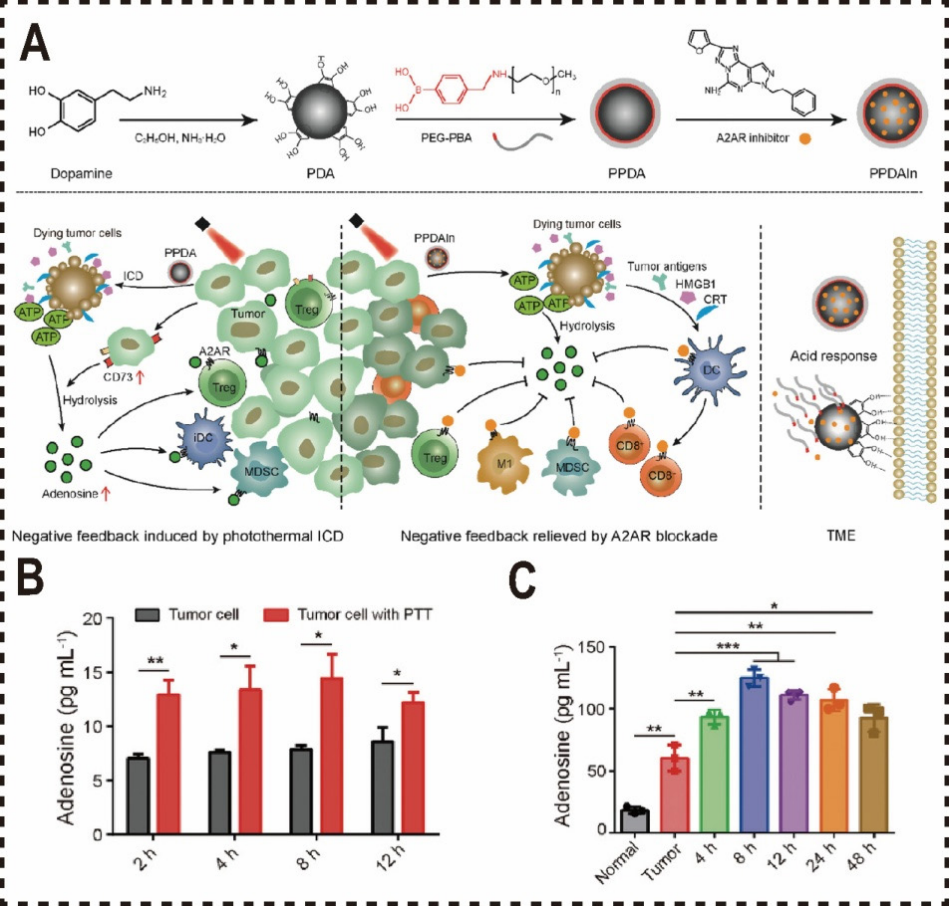
Biomimetic nanomaterials inhibited ADO pathways. (A) Schematic illustration of blocking the metabolic checkpoint A2aR by PPDAIN to enhance ICD immunotherapy efficacy. (B) Adenosine levels in 4T1-cell culture medium after irradiation at 48 °C (n = 3). (C) ELISA detection of tumor adenosine levels after PTT treatment. Normal: normal tissue; Tumor: control tumor without treatment (n = 3). Reproduced with permission from Ref. 183. Copyright 2022, Wiley-VCH.

Beyond utilizing cell membranes as biomimetic materials for the nano-delivery of ADO inhibitors, polydopamine--a biocompatible and biomimetic nanomaterial with robust bioadhesion and photothermal properties, has been widely adopted in formulating PTT combined with ADO pathway blockade. To enhance the water solubility and targeting precision of small molecule ADO antagonists and increase the tumor accumulation of A2aR antagonists, Liu et al. developed an acid-sensitive PEG nanocoating on a polydopamine (PDA) nanoparticle delivery system 183. This system encapsulates the A2aR antagonist SCH58261, enabling tumor-specific delivery and enhancing ICD immunotherapy via PTT. Upon reaching the acidic tumor microenvironment, the PEG shell dissociates, exposing the PDA carrier loaded with the inhibitor. The mussel-inspired adhesive properties of PDA anchor the carrier to tumor tissue, promoting prolonged retention and accumulation at the tumor site. Inhibition of the metabolic checkpoint A2aR mitigates ADO-induced metabolic stress in tumor-infiltrating immune cells and amplifies ICD-mediated effective anti-tumor immune responses. However, the blocking the A2aR pathway frequently encounters challenges including off-target effects and toxicity due to nonspecific drug delivery (**Figure 6A**).

To tackle this challenge, Yu et al. developed a tumor-specific A2AR-blocking nanoparticle delivery system, platinum (Pt)-PDA, integrating the photothermal properties of PDA with the self-activated oxygen delivery capacity of Pt nanoparticles 184. This approach enhances oxygenation at tumor sites through near-infrared (NIR) radiation. Pt nanoparticles emulate CAT to alleviate tumor hypoxia, thereby suppressing the ADO signaling pathway and reducing toxicity linked to A2aR-mediated immunosuppression. Furthermore, NIR radiation enables direct photothermal ablation of tumors, inducing immunogenic cell death and amplifying anti-tumor immunity. Notably, even in immune-resistant triple-negative breast cancer (TNBC) models, this strategy elicited induced robust T cell activation and exhibited significant anti-tumor efficacy.

Furthermore, the application of biomimetic nanomaterials has advanced the clinical utility of specific small interfering RNAs (siRNAs) targeting the ADO signaling pathways. Liu et al. engineered biomimetic nanoparticles camouflaged with B16F10 cell membranes, which were designed to encapsulate CAT and CD73 siRNAs to explore the synergistic effects of hypoxia alleviation and CD73-ADO pathway downregulation on the efficacy of ICB therapy 185. This delivery system efficiently transports CD73 siRNA to target cells, releasing it intracellularly to achieve precise gene silencing. This approach enhances the therapeutic efficacy of siRNA while minimizing off-target effects thereby advancing progress in gene therapy.

### Metal-based nanomaterials

Metal-based nanomaterials (MNMs) demonstrate considerable potential in drug delivery due to their unique metallic properties 186. These materials have been extensively explored as carriers for a diverse array of therapeutic agents, including chemotherapeutic drugs, RNA, antibodies, and peptides 187. MNMs composed of titanium, silver, and gold exhibit tunable optical properties and can be surface-functionalized through hydrogen bonds, electrostatic interactions, and covalent bonds. Enabling the attachment of targeted drugs and bioactive molecules. Moreover, functionalized MNMs can enhance the solubility of hydrophobic drugs, prolong systemic circulation time, mitigate rapid renal clearance, and offer the potential to address multiple therapeutic targets 188. They also facilitate the co-delivery various bioactive compounds alongside imaging agents, supporting targeted delivery for antitumor therapy and diagnostics through surface ligand modifications 189-191. By exploiting the EPR effect, MNMs achieve deep penetration into tumor sites, providing novel research avenues strategies for their combined use with ADO inhibitors.

Compared to other nanomaterials, metal nanomaterials possess distinctive optical, magnetic, and catalytic properties for drug delivery, with their positioning in the body precisely controllable via an external magnetic field. This photo-magnetic combination therapy enables accurate precise drug delivery by synergistically integrating magnetic field guidance and photothermal effects. For example, in tumor therapy, an external magnetic field can direct magnetic MNMs to accumulate at the tumor site, where subsequent light exposure triggers drug release, thereby enhancing therapeutic efficacy. These attributes establish MNMs as a highly promising drug delivery platform. Additionally, the surface of MNMs can be readily functionalized through chemical modifications to conjugate various targeting molecules, such as antibodies, peptides, carbohydrates, or small molecule drugs. These targeting ligands allow MNMs to localize to specific cells or tissues, notably particularly tumor cells or other pathological sites. This capability markedly improves targeting precision and minimizes off-target effects on healthy cells, and reduces side effects when delivering ADO inhibitors, offering a significant advantage over other nanomaterials.

**Figure 7:**
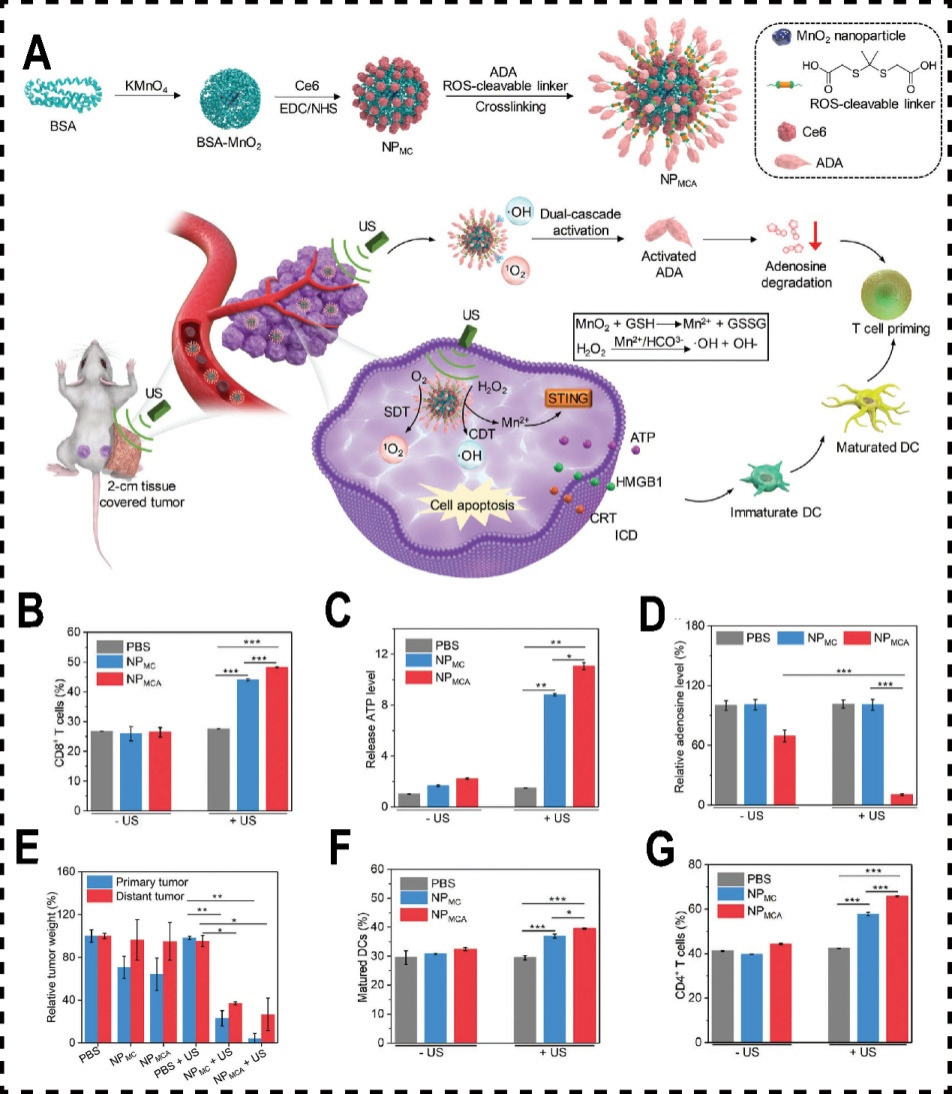
Metal-based nanomaterials inhibited ADO pathway. (A) Schematic diagram of ultrasound (US) and nano-potentiator (NP_MCA_) for CDT and SDT to heighten immunotherapy. (B) Quantitative analysis of ADO release from 4T1 cells in different treatment groups (n = 5). (C) Quantitative analysis of ATP levels within 4T1 cells across different treatment groups (n = 5). (D) Total mass of primary and metastatic tumors across various treatment cohorts (n = 5). (E) The number of mature DCs in different treatment group (n = 5). (F) The number of CD4^+^ T cells in different treatment group (n = 5). (G) The quantity of CD8^+^ T cells across various treatment groups (n = 5). Reproduced with permission from Ref. 194. Copyright 2022. Wiley-VCH.

Inosine, a critical intermediate in purine metabolism, acts as an alternative energy source for Teff under glucose-restricted conditions within the TME 192. It restores T cell proliferation and cytotoxicity, positioning inosine as a vital immune enhance. Adenosine deaminase (ADA) catalyzes the conversion of extracellular ADO into inosine, offering a potential strategy to bolster tumor immune responses by degrading intracellular ADO, thereby preventing its interaction with T cell receptors and inhibiting the ADO pathway 193. However, this approach is hampered by poor selectivity and limited safety. The primary limitation stems from ADA's inadequate capacity to selectively accumulate at tumor sites, compromising its therapeutic efficacy and specificity 194. To overcome these challenges, Xu et al. engineered a dual-stage activatable nanocatalyst conjugate, NPMCA, by conjugating ADA and chlorin e6 (Ce6) to manganese dioxide (MnO₂) nanomaterials. This design employs an ultrasound (US)-responsive nanosystem to achieve precise tumor-specific accumulation of ADA, with controlled release facilitated by reactive oxygen species (ROS)-responsive linker cleavage. In vivo and in vitro experiments demonstrated that MnO₂ nanomaterials respond to endogenous GSH in the TME, decomposing into Mn²⁺ ions that catalyze the conversion of hydrogen peroxide (H₂O₂) into hydroxyl radicals (·OH), enabling chemodynamical therapy (CDT). Simultaneously, Ce6 acts as a sonosensitizer, generating singlet oxygen (^1^O_2_) under ultrasound, mediating mediate sonodynamic therapy (SDT). The ROS generated from the combination of CDT and SDT not only induces ICD in cancer cells but also cleaves the ROS-sensitive linker, achieving dual-stage activation of ADA and significantly enhancing tumor immunotherapy (**Figure 7A**). Additionally, Yu et al developed MnFe_2_O_4_ nanoparticles conjugated with dichloroacetate (DCA) to counteract the tumor's immune-suppressive microenvironment by modulating glucose metabolism and ATP degradation 195. These MnFe_2_O_4_-DCA nanoparticles target mitochondria, releasing oxygen, thereby enhancing DCA bioactivity. They increase glucose metabolism, reduce lactate production, downregulate CD39 and CD73 expression, limit extracellular ATP breakdown, and decrease ADO and lactate levels. This promotes the promotion of DCs maturation and an enhances CTLs responses, ultimately improving cancer immunotherapy outcomes.

In conclusion, we have delineated the roles of various nanomaterial classes in suppressing the ADO pathway (**Table 2**). By integrating with ADO inhibitors or associated enzymes, nanomaterials achieve pathway inhibition either by directly targeting key components such as CD39, CD73, and A2a, or by mitigating hypoxia to curtail ADO production. Each nanocarrier type exploits its distinct properties to overcome the clinical limitations of ADO inhibitors. Consequently, the synergy between nanocarriers and ADO inhibitors offers substantial potential for addressing current obstacles, thereby enhancing the prospects for the clinical translation of ADO inhibitors. This progress could establish the ADO pathway as a cornerstone in cancer immunotherapy.

## Conclusion and outlook

In conclusion, the synergy between nanomaterials and therapeutic agents in suppressing the ADO pathway has garnered substantial scientific attention. This review provides a comprehensive analysis of the immune suppressive mechanisms of ADO, highlights the clinical potential of ADO inhibitors targeting the ADO pathway, and evaluates recent advancements in integrating nanomaterials with ADO inhibitors.

Although numerous preclinical studies have demonstrated potent anti-tumor effects through nanomaterial-mediated delivery of ADO inhibitors, their translation in clinical trials remains limited. For instance, targeting the ADO pathway can result in off-target effects, and the ADO pathway represents a promising therapeutic target for treating a variety of diseases--most notably cancer, cardiovascular disorders, and neurological disorders, this broad applicability complicates precise therapeutic strategies. The primary barriers are as following: (1) Complexity of the ADO Pathway: The ADO pathway involves multiple key molecules, including CD39, CD73, and A2aR, and exhibits synergistic and redundant interactions with other immunosuppressive pathways including PD-1/PD-L1 and TGF-β. This intricacy hinders to substantial alleviation the immunosuppressive state of the TME by targeting the ADO pathway alone; (2) Physiological Roles of ADO: ADO and its associated molecules play critical roles in normal tissues, and disrupting these pathways may trigger to unpredictable side effects, such as cardiovascular dysfunction or systemic inflammation; (3) Hypoxia-Driven Immunosuppression: ADO production is intricately tied to hypoxia within the TME. Even with ADO pathway blockade, hypoxia-induced immunosuppression may persist via alternative mechanisms; (4) Drug Delivery Challenges: Current delivery systems face significant obstacles in achieving precise targeting and controlled release, leading to suboptimal drug utilization and reduced therapeutic efficacy; (5) Lack of Reliable Biomarkers: The absence of validated biomarkers impedes patient stratification and efficacy prediction, diminishing the success rate of clinical trials. Although some preclinical studies and Phase I/II trials have shown encouraging results, these efforts are limited by small sample sizes and a lack of comprehensive long-term data on efficacy and safety. This evidentiary gap hinders widespread clinical adoption of ADO pathway-targeted therapies.

Furthermore, the clinical translation of nanomaterials encounters numerous challenges, representing a primary barrier for the widespread adoption of nanocarrier- mediated ADO inhibitor delivery. The key factors contributing to this limitation can be summarized as follows: (1) Significant differences between murine and human tumors result in notable discrepancies between the preclinical therapeutic outcomes of nanomaterials and their performance in clinical trials; (2) The EPR effects keeps a critical mechanism for nanomaterial accumulation in tumor tissues, but variability in the EPR effects across patients' TME introduces uncertainty regarding the actual accumulation efficiency; (3) The safety and efficacy of nanomaterials cannot yet be fully assured, requiring additional clinical data to validate their performance and ensure that their pose no undue risks to patients. Additionally, the nanomaterials reviewed herein exhibit inherent limitations, including: 1. Lipid nanoparticles: Poor stability and limited drug loading capacity; 2. Micelles: Insufficient targeting precision; 3. Mesoporous silica: Variability particle size, shape, and pore diameter, potentially compromising loading efficiency; 4. MOFs: Susceptibility to degradation under physiological or acidic conditions, diminishing drug delivery efficacy; 5. Biomimetic nanomaterials: Complex design, suboptimal stability, and challenges related to biocompatibility and potential toxicity; 6. Metal-based nanomaterials: Limited biodegradability, potential toxicity, and risks of long-term accumulation in organs such as the liver, spleen, and kidneys, which may lead to systemic toxicity. These obstacles underscore the significant hurdles in translating nanomaterials from laboratory research to clinical practice. Continued research and technological advancements are imperative to address these constraints and promote the broader application of nanomaterials in cancer therapy.

Despite these obstacles, the potential of targeting the ADO pathway in cancer therapy remains substantial. Innovations such as the development of advanced drug delivery systems (e.g., novel biomimetic nanomaterials), the refinement of combination therapy approaches (e.g., synergistic integration with PD-1/PD-L1 inhibitors), identifying highly specific biomarkers, and investigation of interactions between ADO and hypoxic microenvironments could significantly enhance the efficacy and safety of ADO-targeted therapies. Through technological advancements and well-designed clinical trials, targeting the ADO pathway holds considerable promise as a pivotal element of cancer immunotherapy, potentially providing patients more effective therapeutic options.

## Figures and Tables

**Table 1:** Clinical studies of monoclonal antibodies or small molecule ADO pathway inhibitors.

Series	Drug	NCT number	Indications	Phase
CD39 antagonist	TTX-030	NCT03884556	lymphoma	I
CD39 antagonist	SRF617	NCT04336098	PDAC	I
Anti-CD39	ES014	NCT05381935	TNBC	I
Anti-CD39	PUR001	NCT05234853	PDAC	I
CD39 antagonist	ES002023	NCT05075564	NSCLC	I
Anti-CD39	IPH5201	NCT04261075	NSCLC	I
Anti-CD73	JAB-BX102	NCT05174585	PDAC	I
Anti-CD73	GS-1423	NCT03954704	TNBC	I
Anti-CD73	CPI-006	NCT03454451	PDAC	I
CD73 antagonist	AB680	NCT04104672	PDAC	III
Anti-CD73	BMS-986179	NCT02754141	CRC	I/II
Anti-CD73	IBI325	NCT05119998	NSCLC	I
Anti-CD73	PT199	NCT05431270	PDAC	I/II
Anti-CD73	AK119	NCT05173792	CRC, NSCLC	II
CD73 antagonist	MEDI9447	NCT04668300	AS	III
A2aR antagonist	AZD4635	NCT03980821	CRC	II
A2aR antagonist	PBF509	NCT03207867	NSCLC	II
Anti-A2aR	AB928	NCT03720678	mCRC	I/Ib
A2aR antagonist	EOS100850	NCT05117177	SKCM	I
A2aR antagonist	CPI-444	NCT02655822	mCRC	I/Ib
A2aR antagonist	CS3005	NCT04233060	NSCLC	I

**Table 2:** Nanomaterials Synergizing with Various Drugs to Inhibit the ADO Pathway.

Nanomaterials	Application	Characteristics
Lipid nanoparticle	POL 135 RC-nIMs 136	Achieve targeted delivery; Increase the drug load; Reduce the side effects of drugs; Poor membrane stability; Poor targeting specificity for specific cells.
Polymer micelle	CAT-NP 144 D/R@RPsp 145	Long-term blood circulation time; Improved drug stability; Controlled drug release properties; Low loading capacity for hydrophilic drugs; Poor plasma stability.
Mesoporous nanomaterials	D/R/C@SiO_2_ 160 CaP@Fe-MOF 172	Higher drug loading capacity; Reduced drug leakage and dose control; Higher stability; Strong biocompatibility; Poor biodegradability; Limited material options.
Biomimetic nanomaterials	cMLVs 181 PPDAIN 183 Pt-PDA 184	Strong biocompatibility and biodegradability; Strong transmembrane penetration capability; Complex synthesis process; Unstable drug release.
Metal-based nanomaterials	NP_MCA_ 194 MnFe_2_O_4_-DCA 195	Provide photothermal and magnetic combined therapy; Metal toxicity.

## Abbreviations

ATP: Adenosine triphosphate
CD39: Ectonucleoside triphosphate Diphosphohydrolase-1
CD73: Extracellular 5'-nucleotidase
CD80: Cluster of Differentiation 80
CD86: Cluster of Differentiation 86
TME: Tumor microenvironment
ICD: Immunogenic cell death
HIF-1: Hypoxia-inducible factor 1
TGF-β: Transforming growth factor β
DCs: Dendritic cells
MDSCs: Myeloid-derived suppressor cells
Tregs: Regulatory T cells
MHC: Major histocompatibility complex
IL-2: Interleukin-2
IL-4: Interleukin-4
IL-5: Interleukin-5
IL-6: Interleukin-6
IL-8: Interleukin-8
IL-10: Interleukin-10
IL-12: Interleukin-12
IL-17: Interleukin-17
TNF-α: Tumor necrosis factor-α
VEGF: Vascular endothelial growth factor
CTL: Cytotoxic T lymphocyte
APCs: Antigen-presenting cells
TCRs: T cell receptors
PK-70: Protein kinase-70
cAMP: Cyclic adenosine monophosphate
PKA: Protein kinase A
Teffs: Effector T cells
IL-2R: Interleukin-2 receptor
LCK: Lymphocyte-specific protein tyrosine kinase
CREB: Cyclic-AMP response binding protein
NF-κB: Nuclear factor kappa-B
CXCL9: C-X-C motif chemokine ligand 9
CXCL10: C-X-C motif chemokine ligand 10
IFN-γ: Interferon-γ
PD-1: Programmed cell death 1
CTLA-4: Cytotoxic T lymphocyte associate protein-4
GM-CSF: Granulocyte-macrophage colony-stimulating factor
MIP-1α: Macrophage inflammatory protein-1 alpha
PI3K-PKC-HIF1: Phosphoinositide 3-kinase-Protein kinase C-Hypoxia-inducible factor
MAPK-AP-1: Mitogen-activated protein kinase- activator protein-1
EPAC-p38-C/EBP: cAMP-p38 MAPK-CCAAT/enhancer-binding protein
MDS: Myelodysplastic syndromes
Gal-9: Galectin-9
LAG-3: Lymphocyte Activation Gene-3
PD-L1: Programmed cell death 1 ligand 1
CAR T: CAR-engineered T cell
RCC: Renal cell carcinoma
PDAC: Pancreatic ductal adenocarcinoma
TNBC: Triple- negative breast cancer
CRC: Colorectal cancer
mCRC: Metastatic colorectal cancer
AS: Angiosarcoma
SKCM: Skin cutaneous melanoma
CD107a: Lysosomal-associated membrane protein 1
RT: Radiotherapy
CT: Chemotherapy
EPR effect: Enhanced Permeability and Retention effect
PEG: Polyethylene glycol
PVP: Polyvinylpyrrolidone
pNIPAM: Poly(N-isopropylacrylamide)
DOX: Doxorubicin
TLR7/8: Toll-like receptor 7 and 8
GSH: Glutathione
ICB: Immune checkpoint blockade
MOFs: Metal-organic frameworks
NMOFs: Nanoscale MOFs
R837: Imiquimod
UV: Ultraviolet
R848: Resiquimod
ADK: Adenosine kinase
AMP: Adenosine monophosphate
BPQD: Black phosphorus quantum dots
AMPCP: α, β-methylene adenosine 5 '-Diphosphate
PTT: Photothermal treatment
CAT: Catalase
ADA: Adenosine deaminase
ROS: Reactive oxygen species
CDT: Chemical dynamic therapy
SDT: Sonodynamic therapy
MNMs: Metal nanomaterials
NSCLC: Non-small cell lung cancer
